# Artificial Intelligence–Based Mobile Phone Apps for Child Mental Health: Comprehensive Review and Content Analysis

**DOI:** 10.2196/58597

**Published:** 2025-06-06

**Authors:** Fan Yang, Jianan Wei, Xuejun Zhao, Ruopeng An

**Affiliations:** 1 School of Social Work, University of Illinois Urbana Champaign, Urbana, IL, United States; 2 School of Public Administration, Dongbei University of Finance and Economics, Dalian, China; 3 School of Computer Science and Engineering, Macau University of Science and Technology, Macau, China; 4 Constance and Martin Silver Center on Data Science and Social Equity, Silver School of Social Work, New York University, 726 Broadway, New York, NY, 10003, United States, 1 2129981212

**Keywords:** mental health, children, artificial intelligence, mobile health, AI-driven mobile applications, mobile phone

## Abstract

**Background:**

Mobile phone apps powered by artificial intelligence (AI) have emerged as powerful tools to address mental health challenges faced by children.

**Objective:**

This study aimed to comprehensively review AI-driven apps for child mental health, focusing on their availability, quality, readability, characteristics, and functions.

**Methods:**

This study systematically analyzed AI-based mobile apps for child mental health. Quality was evaluated using the Mobile Application Rating Scale, which assessed various dimensions of app quality, including subjective quality, engagement, functionality, aesthetics, and information. An automatic readability index calculator was implemented to assess readability by using the count of words, syllables, and sentences to generate a score indicative of the reading difficulty level. Content analysis was conducted to examine the apps’ availability, characteristics, and functionality.

**Results:**

Out of 369 apps initially identified, 27 met the eligibility criteria for inclusion. The quality of the apps was assessed using Mobile Application Rating Scale, with an average score of 3.45 out of 5 (SD 0.5), indicating a need for quality improvement. The readability analysis revealed suboptimal scores, with an average grade level of 6.62 (SD 2.2) for in-app content and 9.93 (SD 2.6) for app store descriptions. These results, combined with a monotonous user interface, suggest that many apps lack a child-friendly design, potentially hindering their usability and engagement for young users. Content analysis categorized the apps into 3 functional groups—chatbot-based apps (15 apps), journal logging apps (9 apps), and psychotherapeutic treatment apps (3 apps). While 20 out of 27 apps (74%) used clinically validated technologies, rigorous clinical tests of the apps were often missing, with only 2 apps undergoing clinical trials. Of the 27 apps analyzed, only 7 (26%) were free to use, while the majority, 20 apps, required a subscription or one-time payment. Among the paid apps, the average cost was US $20.16 per month, which may pose a financial barrier and limit accessibility for some users, particularly those from lower-income households.

**Conclusions:**

AI-based mental health apps hold significant potential to address the unique challenges of child mental health but face critical limitations in design, accessibility, and validation. To fully realize their benefits, future research and development should focus on integrating child-centric design principles, ensuring affordability, and prioritizing rigorous clinical testing. These efforts are essential to harness the power of AI technologies in creating equitable, effective, and engaging solutions for improving child mental health outcomes.

## Introduction

Mental health, as defined by the World Health Organization, is a state of well-being where individuals effectively manage stress, recognize their abilities, work productively, and contribute to their community. It includes conditions such as mental disorders, psychosocial disabilities, and states involving significant distress or functional impairment [1]. Concerns regarding mental health, particularly among children—defined as individuals younger than 18 years of age [2]—have garnered increasing attention in recent years. Around the world, 1 in 7 children experiences mental health conditions, yet many more remain largely unrecognized and untreated [1]. Children with mental health challenges face various risks, including social exclusion, discrimination, stigma, educational difficulties, risk-taking behaviors, physical health symptoms, and human rights violations [3]. To safeguard their lifelong health and well-being, it is crucial to shield children from adversity, promote psychological wellness, and ensure their accessibility to mental health care [4,5].

Many children grapple with mental health issues, and an overwhelming fraction encounter barriers to accessing effective, affordable, and high-quality mental health services [6]. Several factors contribute to this limited access, including lack of health insurance, inadequate financial support, cultural constraints, and extended waiting times due to a shortage of professional services [4]. Beyond these microlevel challenges, public health crises, notably the COVID-19 pandemic, have exacerbated disparities between the escalating mental health needs of children and the availability of quality care. During the pandemic, national surveys across various countries revealed a heightened mental health strain on children, exacerbated by lockdowns that limited access to services and reduced help-seeking out of fear of contracting virus [1,7,8].

The swift advancement of technology, particularly in communication, has paved the way for novel mental health support mechanisms. The widespread availability of mobile phones and associated apps makes it possible for individuals to easily tap into essential resources, irrespective of where they are [9]. This democratization of access has been particularly impactful for mental health services, which are now available at the fingertips of those in need, often at a fraction of the traditional cost [10]. The integration of artificial intelligence (AI) in these apps has further revolutionized the landscape. As noted by Boucher et al [11], the infusion of AI technology not only enhances the precision of these platforms but also imbues them with a more empathetic and human touch. This allows for a tailored intervention, optimizing the support offered based on an objective analysis of user data.

Growing up in the digital era, children are inherently familiar with digital devices such as smartphones and tablets [12,13]. These tools offer an intuitive and user-friendly interface, eliminating complexities or abstract concepts and reducing potential barriers for younger users [13]. Previous research highlights a strong preference among children and adolescents for AI-integrated tools [13]. Studies suggest that AI-informed mobile health apps hold significant potential in addressing mental health issues in children [14]. These apps are generally well-received, with children showing positive attitudes toward their integration. Experts view such tools as complementary to traditional in-person interventions, offering low-threshold access via smartphones and extending their reach to children in high-risk situations or underserved communities [14]. This trend underscores the readiness of these age groups to embrace and engage with emerging technologies.

While AI-powered mental health apps are increasingly prevalent in the market, concerns have been raised about their effectiveness and quality [15,16]. These concerns are heightened by the rapid pace of AI advancement and evolving mental health care guidelines. It is important to note that most of the current scientific literature concentrates on adult mental health, often overlooking the unique needs of children. Recent comprehensive reviews by Li et al [17] and Hua et al [18] have made significant contributions to the field by providing valuable insights into AI-based conversational agents and large language models, respectively. Our study builds upon this foundation by encompassing a diverse array of AI-driven mobile phone apps, both those exclusively designed for children and those that include children as part of their broader target audience. This includes not only conversational agents, but also AI techniques used for emotion and journal tracking, as well as psychotherapeutic treatment within mental health apps. Furthermore, while these previous works have addressed AI applications in a general population context, our research uniquely targets the mental health of children, a demographic with specific needs and challenges that are often underrepresented in the literature. By systematically reviewing AI-based mental health apps accessible to children, our study aimed to highlight the current gaps and challenges in the development of child-specific resources within this emerging field. We evaluated 5 core areas—availability, quality, readability, characteristics, and functionality. Our insights aim to lay the groundwork for future research and guide the creation and development of AI-driven mental health apps that better cater to the needs of children, ultimately fostering the long-term psychological well-being of our future generations.

## Methods

This study aimed to systematically review AI-driven mobile apps designed for children’s mental health. The review process involved multiple steps, including a thorough literature search, screening, and evaluating selected apps.

### App Search and Screening Strategy

An extensive search was conducted in the 2 most widely used marketplaces for mobile phone apps: Android’s Google Play Store and Apple’s iOS App Store. Search terms included “AI mental health apps for child/children” and “AI mental health apps for teenagers.” These apps were subsequently screened based on the following criteria: (1) available in English; (2) can be downloaded for current use from Google Play or the Apple App Store; (3) the primary function is improving mental health; (4) the app must explicitly state that it is AI-based, with the AI component being central to its functionality in addressing mental health issues; and (5) the minimum age of the user shall be less than 18 years old.

The minimum age for app users in app stores is determined by a combination of factors, including developer-provided information, content ratings, and compliance with legal regulations. Developers are required to disclose the intended audience and the highest age-rated content available in their apps, which is then aligned with standardized rating systems like those developed by the International Age Rating Coalition. These systems ensure that apps are categorized appropriately based on their content, features, and potential risks. Legal frameworks, such as the Children’s Online Privacy Protection Act in the United States and the General Data Protection Regulation in the European Union, further influence these age ratings by setting strict guidelines for data collection from children, thereby shaping the minimum age requirements [19]. Using the app stores’ minimum age ratings as a criterion for this study ensured that the reviewed apps include both those explicitly designed for children and those that, while not exclusively targeting children, include children as part of their broader user population. This dual inclusion provides a comprehensive analysis of AI-based mental health support tools available to children aged less than 18 years.

Apps that did not meet these criteria were filtered out. Furthermore, 2 research investigators (JW and XZ) independently conducted this screening process and reached a consensus on the final selection.

### App Evaluation

Two investigators (JW and XZ) independently evaluated the apps. The Mobile Application Rating Scale (MARS) was primarily used to assess the quality of the apps, while a readability calculator was used to evaluate the readability. Content analysis was conducted to determine the descriptive characteristics and functionality. Descriptive characteristics codes included (1) price (free vs for-purchase); (2) confidentiality, concerning whether the app communicated and managed policies related to user privacy and data security; and (3) AI techniques adopted, referring to specific AI techniques used by the apps. Mobile app functionality components were also evaluated, including (1) clinically-proven psychotherapeutic treatment, detailing whether the app’s treatments or the app itself have been clinically validated; (2) target mental health problems about specific mental health issues the app was designed to address; and (3) functions. Any disagreements about an app were resolved through discussion until a consensus was reached.

### Readability of Mobile Apps

The readability of the mobile apps was assessed by 2 independent researchers (JW and XZ) using an automatic readability index calculator. Considering the prevalence of children’s apps being downloaded by parents but primarily used by the children themselves [20], our analysis strategically separated the readability assessment into 2 distinct parts—examining the descriptions listed in the app store and the in-app text. For the in-app text, all visible text on the app pages during typical use was included in the analysis. If a page contained minimal text insufficient for readability evaluation, further interaction with the app was conducted to generate additional text output, such as engaging with chat features. Unrelated content, such as advertisements, was excluded to focus on text that directly impacts user interaction and comprehension. The Flesch-Kincaid Grade Level, an integral component within the readability calculator, was selected as our standard for readability, given its widespread recognition within the field [21-23]. The readability score was calculated using the formula below. The index corresponds to the US elementary school grade levels associated with the evaluated text, offering a universally comprehensible measure of text readability. A higher value on the readability index denotes a higher grade level, implying that more advanced reading proficiency is required.


$$Readability=0.39*\left(\frac{total\;number\;of\;words}{total\;number\;of\;sentences}\right)+11.8*\frac{total\;number\;of\;syllables}{total\;number\;of\;words}-15.59$$

### MARS Assessment

The quality of the apps was evaluated using MARS, a widely recognized standardized quality measurement tool for health-related mobile apps [23-26]. MARS was characterized by 2 principal dimensions—the subjective quality scale, which imparted valuable insights into user experiences and formed a crucial part of the comprehensive evaluation index, and the objective scale, which provided a rigorous, structured evaluation of the apps’ various technical and functional attributes. The objective scale comprised 19 items rated on a 5-point scale (1=inadequate, 2=poor, 3=acceptable, 4=good, and 5=excellent). The items within the objective scale were further divided into 4 thematic categories: engagement, functionality, aesthetics, and information*.* Each category assessed diverse elements, from user interactivity and design appeal to the accuracy and credibility of the information provided. The final MARS score was the mean of the 4 subscores in these dimensions. This structured approach accomplished a comprehensive and detailed evaluation of the app’s quality.

### Content Analysis

In addition to evaluating the apps by the readability index calculator and the MARS scale, we also used content analysis to assess the included apps. Content analysis encompassed 3 main areas—availability, the descriptive characteristics of the apps, and functionality. Availability evaluated the presence of the apps across various app stores. Descriptive characteristics codes included (1) price (free vs for-purchase); (2) confidentiality, concerning whether the app communicated and managed policies related to user privacy and data security; (3) AI techniques adopted, referring to specific AI techniques used by the apps; and (4) children-specificity. Mobile app functionality components were also recorded, including (1) clinically-proven psychotherapeutic treatment, detailing whether the app’s treatments or the app itself have been clinically validated; (2) target mental health problems about specific mental health issues the apps were designed to address; and (3) functions.

Two coders (JW and XZ) underwent training to attain reliability across several rounds of sample mental health apps designed for children. They were required to achieve an interrater reliability criterion of K=0.80 on 10 independently coded apps before commencing coding on the included apps in this study [27]. The 27 apps were downloaded from their respective app stores, and each was assessed and coded by these 2 coders. An overall interrater reliability of 0.94 was achieved, indicating strong reliability [28].

## Results

### Overview

**Figure 1.**
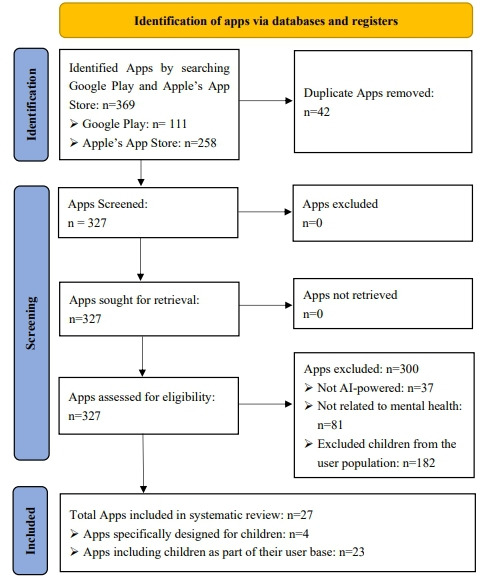
PRISMA (Preferred Reporting Items for Systematic Reviews and Meta-Analyses) of app search and screening strategy. AI: artificial intelligence.

As of December 2024, this search yielded 369 apps. After removing 42 duplicated apps, 327 apps remained. The 327 apps were evaluated based on the inclusion criteria. This selection process resulted in 27 apps being included in this review study, with 4 apps specifically designed for children and 23 apps included children as part of their broad user base. The detailed selection process was demonstrated in Figure 1; the PRISMA checklist has been provided (Checklist 1).

### Readability

Our study used the Flesch-Kincaid Grade Level index in a readability calculator to evaluate the readability of the app descriptions in the app store and in-app contents. The test revealed that the content readability within the apps spanned from grades 3 to 11, with an average readability of grade 6.62 (SD 2.2). This suggested a certain threshold of readability for younger children. The readability of the app description in the app store had a mean grade level of 9.93 (SD 2.6), which was notably higher than the content’s readability. This could be due to the anticipation of parental involvement during the app download and installation processes, as parents are generally expected to have higher reading proficiency and are thus more likely to comprehend such introductions.

### Quality Evaluation

**Figure 2.**
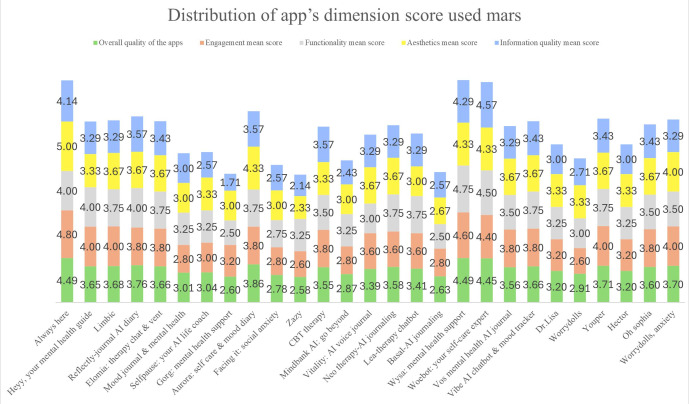
Total MARS scores and subscores across 4 dimensions. MARS: Mobile Application Rating Scale.

Our findings revealed a suboptimal overall quality among existing apps, with MARS scores varying from 2.58 to 4.49, with an average of 3.45 (SD 0.5) on a scale of 1 to 5. Figure 2 employed a stacked bar graph to delineate the subscores and the composite MARS scores across these 4 dimensions. Of particular concern was the information quality, which had the lowest mean score of 3.19 (SD 0.6) and a minimum of 1.71. In contrast, engagement scored the highest, with a mean of 3.56 (SD 0.6) and a score range of 2.6‐4.8.

The Engagement subscale emerged as the highest-scoring category in our study. This subscale was assessed through 5 perspectives*—*customization, interactivity, entertainment, interest, and target group. Delving deeper into the results, 44% (12/27) of the apps incorporated a baseline log-in assessment, emphasizing user customization, while 56% (15/27) did not include this feature. The apps displayed varied engagement strategies; 56% (15/27) primarily used dialogue interactions, and 33% (9/27) focused on diary log inputs. However, many apps needed more rigorous tracking and oversight mechanisms, suggesting a gap in ensuring long-term user interactivity. In addition, the apps exhibited diverse levels of entertainment and interest, with scores ranging from 1 to 5. These variances, especially in entertainment and interest, could affect the target group’s appropriate level.

In total, 4 primary characteristics, performance, ease of use, navigation, and gestural design, were used to assess functionality. In terms of performance, 7 (26%) apps out of 27 demonstrated low accuracy or speed in their features and components, such as buttons and menus, adversely affecting the user’s experience. Ease of use was apparent in 24 apps out of 27 (89%), as they facilitated effortless use for users with distinct menu labels and instructions. When evaluating navigation, these apps generally exhibited a coherent and uninterrupted transition between screens, with essential links in place. The scale score of this component ranged from 3 to 5, with an average score of 3.41 (SD 0.5), indicating that the apps’ navigation is relatively well-integrated and logically structured. In the domain of gestural design, the apps consistently exhibited intuitive user interactions, such as tapping, swiping, pinching, and scrolling, across various components and screens. The evaluation scores for this aspect ranged from 2 to 5, with an average score of 3.48 (SD 0.6).

In evaluating the aesthetic appeal of the apps, particularly given their primary target audience of children, our findings revealed some limitations in their overall design. Based on the aesthetic subscale’s criteria – which included layout, graphics, and visual appeal – only 11 (41%) of the reviewed apps showcased vibrant and engaging designs that effectively captivated the intended population. In stark contrast, 56% (15/27) of the apps exhibited monotonous designs that, while functional, lacked the visual allure necessary to maintain the interest of a younger audience. This aspect was particularly evident in the layout and graphics, where the arrangement and quality of buttons, icons, menus, and content were often not visually appealing. Especially in the visual appeal part, the score range was 2‐5, and most ratings were on the lower end, indicating that the overall appeal of the app design was not strong. Furthermore, 15% (4/27) of these apps predominantly featured dark color schemes, which could potentially create negative psychological impressions and detract from their attractiveness to children. This mismatch between the apps’ aesthetic elements and their intended users’ preferences highlighted a significant area for improvement in app design.

In assessing the apps from an information perspective, our findings demonstrated high accuracy in describing features, aligning well with the first characteristic of the information subscale. Nearly all apps accurately reflected their related features in app stores, avoiding exaggeration or misinformation. Regarding the goals, the majority of the apps were explicit in stating specific, measurable, and achievable objectives. Regarding the quality of information, 68% (18/27) of the apps, primarily developed by research laboratories and scientific institutions, provided correct, well-written content relevant to their goals. This underlined their credibility as sources of information. Regarding quantity, most apps offer comprehensive yet concise information within the scope of their intended use. In addition, the visual information in these apps generally presented concepts clearly and logically through various means like charts, graphs, images, and videos.

### Content Analysis

Content analysis systematically evaluated app availability, descriptive characteristics, and functionality (Table 1). In terms of app availability, all apps were sourced from the Apple App Store and Google Play Store. There were 22 apps available in both app stores. Furthermore, 4 apps were exclusively available in the Apple App Store, and 1 was solely offered in Google Play.

**Table 1.** Content analysis for availability, descriptive characteristics, and functionality.

Analysis criteria	Subcategory	Value, n (%)
Availability		
Availability	Google Play	1 (4)
Availability	Apple’s App Store	4 (15)
Availability	Both Google and Apple	22 (82)
Descriptive characteristics		
Cost	Free	7 (26)
Cost	Purchased	20 (74)
Confidentiality	Yes	27 (100)
Confidentiality	No	0 (0)
AI techniques	Natural language processing	20 (74)
AI techniques	AI-based recommendation system	2 (7)
AI techniques	Automatic speech recognition	1 (4)
AI techniques	Data analysis and tracking	7 (26)
Children specificity	Yes	4 (15)
Children specificity	No	23 (85)
Functionality		
Clinical-proven	Yes	25 (93)
Clinical-proven	No	2 (7)
Psychotherapeutic treatment	None	7 (26)
Psychotherapeutic treatment	Cognitive behavioral therapy	19 (70)
Psychotherapeutic treatment	Dialectical behavior therapy	4 (15)
Psychotherapeutic treatment	Acceptance and commitment therapy	1 (4)
Psychotherapeutic treatment	Exposure therapy	1 (4)
Target problems	General	21 (78)
Target problems	Anxiety, depression, and stress	6 (22)
Functions	Dialogue interaction	15 (56)
Functions	Emotion and journal tracking	9 (33)
Functions	Psychotherapeutic treatment	3 (11)

We have identified critical, descriptive characteristics of these apps in terms of cost, confidentiality, AI techniques, and children’s specificity. There were 20 apps requiring payment to access additional content, with prices ranging from US $2.99 to US $120 per month (mean US $20.16), while only 7 apps offer basic functionality for free. All apps ensured privacy protection by including privacy policies in the App Store and Google Play. AI technologies include natural language processing (NLP), AI recommendation systems, automatic speech recognition, and data analysis and tracking. NLP was used in 74% (20/27) of the apps, mainly for chatbot interactions, while automatic speech recognition was adopted in 1 voice input app. Furthermore, 9 emotion and journal tracking apps used data analysis to monitor and track users’ mental health. Although all the apps have age limits that make them accessible to children, most (23/27, 85%) were not explicitly designed for a young audience, often lacking content and interfaces tailored to be child-friendly. Only a small proportion (4/27, 15% apps) were explicitly tailored for children, featuring age-appropriate content and user interfaces.

Functionality components were evaluated from the perspectives of clinically proven psychotherapeutic treatment, targeting mental health problems, functions, and challenges. Despite the utilization of clinically validated psychotherapeutic treatments like cognitive behavioral therapy and dialectical behavior therapy in 74% (20/27) of the apps, the overall effectiveness of the apps largely remained untested, with 2 apps, Woebot developed by Woebot Health and Youper developed by Youper Inc, having undergone clinical trials. Woebot underwent a clinical trial involving 36,070 participants, which revealed that users of the app experienced a significant reduction in depression and anxiety symptoms after 3‐5 days of use [29]. Similarly, Youper has shown clinical evidence of benefit in a study involving its paying users. This study found that Youper was effective in reducing symptoms of anxiety and depression, with significant improvements observed [30]. Although this provides promising support for both Woebot and Youper, further rigorous testing, particularly through additional randomized controlled trials, is needed to establish their clinical effectiveness more definitively. In terms of targeted mental health problems, 22 apps focused on general mental health problems, while 5 apps were explicitly designed to treat anxiety and depression. Included apps can be divided into 3 major categories based on functions—dialogue interaction, journal log tracking, and psychotherapeutic interventions, which accounted for 56% (15/27), 33% (9/27), and 11% (3/27), respectively.

The utilization of these apps revealed several challenges, which can be categorized into 3 primary areas. Initially, a significant concern was the reliance on static large language models, which do not update their parameters after deployment and therefore may not align with rapid advancements in the field. This led to 12 apps needing help with complex or nuanced user inputs, resulting in ineffective or delayed processing. The responses of 7 apps tended to be repetitive and needed help to deliver meaningful conversation after multiple trials of conversation or when topics extended beyond standardized themes. Second, 9 apps exhibited a critical deficiency in user stickiness and feedback mechanisms. User stickiness was evaluated by examining factors including the app’s ability to re-engage users after periods of inactivity and the presence of features that incentivize continuous interaction, such as personalized content, reminders, and progress tracking. The lack of robust tracking and feedback systems may precipitate user attrition, diminishing the apps’ potential advantages. The third and equally significant challenge was observed in 4 apps, particularly concerning their approach to providing clear guidance. Furthermore, 1 app showed inconsistent dialogue transitions, where choices made by users were not appropriately reflected in the following responses, leading to confusion.

In addition, the game elements within one app needed explicit instructions, creating usability challenges for young users. Explanations or instructions were absent from the results displayed on the card surface, making it difficult for children to comprehend. Collectively, these challenges resulted in a less intuitive and supportive user experience, highlighting the need for more dynamic and user-centric design in these apps.

## Discussion

### Summary of Findings

In an era characterized by the rapid progression of AI, an increasing interest in leveraging technological advancements to address population-level mental health care needs has emerged [27]. In the context of growing societal attention to child mental health challenges [4], our research assessed the quality, readability, characteristics, and limitations of AI-driven mental health apps oriented to children. We identified 3 main functional categories—chatbots (15 apps), journal logging (9 apps), and psychotherapeutic treatment (3 apps), with 74% (20/27) employing NLP technology. The average MARS score of 3.45 out of 5 (SD 0.5) indicated a need for quality improvement. Readability was not sufficiently child-friendly. While most apps (25/27, 93%) used clinically validated technologies, rigorous clinical testing was lacking, with only 2 apps having undergone clinical trials. In addition, concerns about response accuracy and the high costs associated with these apps—74% (20/27) required payment, averaging US $20.16 per month—could limit their accessibility and effectiveness for children.

### Underrepresentation of Child-Specific Design

Empirical research has underscored a positive inclination among children and adolescents toward AI and its integration with interventions, suggesting a solid willingness within these age groups to explore these technologies [13]. However, the evident lack of child-centric design may undermine these potential advantages, as identified in our investigation.

In the context of readability, these apps primarily required advanced reading skills. Previous studies have shown that parents are often the gatekeepers of the app download and installation process. At the same time, children are the users [31]; we evaluated the readability of app descriptions in the app store and content separately. Our findings correspond with the broader readability challenges of mobile apps designed for vulnerable populations [23]. The readability of user interface content—consistently 2‐4 grade levels lower than the app descriptions—still presented a considerable readability threshold for younger users, raising the accessibility barrier for app usage and potentially hindering independent use by children [4].

The content incorporated in these apps was not explicitly tailored to children. For example, our findings revealed that certain apps primarily focused on alleviating occupational stress and addressing professional interpersonal conflict, thereby falling short of addressing the unique mental health requirements of children. This mismatch can be attributed to 2 key factors. First, due to the limited availability of apps exclusively designed for children’s mental health, our study included apps that, while not specifically targeted at children, were accessible to them within the broader age range intended by the developers. As a result, some apps that children could access contained content not relevant to their specific needs. Second, this mismatch also reflects the developers’ limited understanding of children’s mental health needs, leading to the inclusion of content that does not align with children’s requirements. App developers were predominantly technology firms and research laboratories without professional child mental health experts to inform app design. Hence, based on the recommendations made by previous studies [13], we advocated for a multidisciplinary team, comprised of AI and app technicians alongside child mental health professionals, to guide the future development of AI-driven apps designed to address children’s mental health concerns. This approach should involve collaboration between AI and app developers, child mental health professionals, and endorsement by peak mental health organizations. The involvement of these key stakeholders ensures that the apps are clinically effective, relevant to the target population, and endorsed by authoritative bodies, thereby increasing their credibility and acceptance in clinical practice. Furthermore, the cocreation of these apps with children and clinicians can enhance their usability and ensure that they meet the specific needs of the target population.

The intervention delivery method within these apps also demonstrated a need for more child-friendly techniques. Diary-logging apps and chatbot dialogues were the dominating intervention strategies employed by these apps. However, diary logs and chatbot interactions needed substantial text input and the ability for users to clearly articulate their mental health concerns, an approach that has been evidenced as being more challenging for child users [4,27]. The expressive capabilities of children may need to be sufficiently developed to leverage these apps, thereby impacting their effectiveness. Incorporating child-friendly tools such as art therapy [4], an area made achievable by recent developments in text-to-image models like stable diffusion models and DALL·E, could offer potential solutions.

In addition to readability, app content, and intervention delivery method, the existing apps’ visual aesthetics and user interface design also demonstrated a need for child-centric considerations. The color palette of many current apps could have been more varied, simplistic, and potentially unengaging, lacking vibrant imagery that could capture children’s attention [32]. On the contrary, some apps adopted a dark aesthetic, which could appear somber and oppressive, potentially intensifying feelings of discomfort or even burden, particularly in users already experiencing low mood or mental health issues [33]. Hence, it was advisable for app designs to integrate richer, brighter colors and more engaging images. Including popular animation characters could enhance children’s interest and engagement [4].

### Barriers to Optimal Mental Health Outcome Concerns

To effectively embed the findings of this study into clinical practice, it is crucial to integrate AI-driven mental health apps into existing care frameworks. This integration can enhance the accessibility and quality of mental health care for children, particularly in underserved populations. However, for these tools to be successfully adopted in clinical settings, their effectiveness in producing measurable behavior change and improving mental health outcomes must first be validated. While our review highlights the potential of AI-driven mobile phone apps in addressing child mental health, it is important to note that the effectiveness of these interventions in producing measurable behavior change remains largely untested. Therefore, future research should focus on evaluating the impact of these apps on users’ mental health outcomes and behavioral modifications. Establishing this evidence is essential to ensure that these apps can be confidently and effectively incorporated into routine care, ultimately delivering the intended benefits to the target population.

The effectiveness of an app was fundamentally linked to the therapeutic technology it employed [27]. Most reviewed mobile apps used clinically validated technologies, such as cognitive behavioral therapy and dialectical behavior therapy [34]. The effectiveness of an app was fundamentally linked to the diagnostic and therapeutic technology it employed [31]. However, these apps’ effectiveness in enhancing child mental health largely remained clinically unproven, a conclusion drawn from our review of 27 such apps. Even though these apps primarily serve adjunctive support tools and cannot fully replace professional psychological diagnosis and treatment [11], we strongly recommend that these apps undergo rigorous clinical validation to ensure their effectiveness, underpinned by solid evidence.

User engagement and stickiness are critical factors that can influence the effectiveness of mental health interventions. The journey toward mental health improvement is cumulative, requiring consistent commitment over time [11]. This underscores the importance of regular app usage and the fostering of robust user engagement for optimal intervention functionality [35]. However, a significant shortcoming in the design of most current apps is the absence of effective user tracking mechanisms. User tracking mechanisms were examined by examining the presence of features such as usage analytics, personalized reminders, and progress tracking, which are essential for promoting regular interaction. These features are known to encourage regular use and engagement, which are important for the success of any intervention [36-38]. Several apps have implemented systems requiring notification activation to promote more regular user interaction [11], contributing to a more effective mental health intervention experience.

Furthermore, the interplay between AI-powered mental health apps and user privacy presented unique strengths and challenges that can indirectly affect mental health outcomes by altering the users’ decisions on whether to engage with these apps. The stigma attached to mental health issues in various cultural and societal contexts heightens privacy concerns [31], especially among underrepresented populations with limited access to formal mental health care [39]. AI-powered mobile phone apps can positively enhance the sense of privacy credited to no human involvement [40]. However, chatbot dialogues and journal logging may involve significant exposure of personal and traumatic experiences, raising critical privacy concerns about handling such sensitive data [10]. This potential exposure may deter many privacy-sensitive individuals from using these apps. To alleviate these concerns, many apps have adopted privacy safeguards such as presenting privacy policies during initial installation, anonymizing data, and postsession deletion of user histories. Transparency in implementing and communicating these privacy-related strategies remained crucial.

The association between low socioeconomic status and adverse child mental health outcomes has been widely described [8,41-43]. On the flip side, cost often presents a significant obstacle in accessing mental health services, with affordability issues preventing nearly half of the individuals requiring mental health treatment in the United States from receiving it [44]. In this context, the presumed high accessibility of AI-powered mental health apps, both in academia and public perception, was considered a significant advantage, fostering wider user reach and coverage [32]. However, most apps we evaluated incorporate fee-based features, raising the barrier to use. This increased cost compromises the perceived accessibility of the AI and mobile app approach, potentially curtailing its effectiveness.

### Challenges and Considerations in AI Techniques for Child-Oriented Mental Health Apps

The current landscape of AI in child-oriented mental health apps reveals a significant reliance on NLP to assess and intervene in users’ mental health states. While NLP is a powerful tool for processing and analyzing language, this predominant focus on text-based interactions may not fully address the needs of children, who often struggle with limited reading, verbal, and writing skills, particularly when expressing complex mental health concerns. The lack of diverse interaction methods could hinder children’s ability to engage with and benefit from these mental health interventions. In light of this, we suggest future research and apps incorporating child-friendly AI techniques, such as generative AI for image creation using natural language prompts, to complement NLP-based approaches. Image-based tools can help children express their emotions and thoughts more effectively, broadening the accessibility and impact of mental health apps for this demographic. By offering multiple modes of interaction, these apps could better accommodate the diverse needs and abilities of children, ultimately making them more effective tools for promoting mental health among younger users [3].

Consistent with previous research [10,11,40,45], our findings revealed that the quality of the AI techniques currently in use raised considerable concerns. The performance of NLP, in particular, demonstrated notable inconsistencies and disparities among different mobile phone apps. For instance, some apps needed help processing simple questions, often responding with repetitive and uninspiring answers after prolonged periods. This issue was especially evident when voice input—an interaction mode that naturally resonates with and is easily used by children [5]—was used. These apps’ inability to handle such a fundamental form of interaction effectively raised significant questions about their usability and overall quality [46]. Given these limitations, users might be deterred from relying on these tools for consistent support, limiting their potential benefits.

In light of these quality issues, we suggest that the application of implementation science principles is vital for the practical deployment of AI-driven mental health apps. One approach is for developers to prioritize incorporating state-of-the-art language models. One of the key challenges in this context is preventing AI hallucinations—where AI generates inaccurate or misleading information. To mitigate this risk, we propose linking AI systems within these apps to established clinical guidelines using advanced information retrieval methods. This approach ensures that AI provides evidence-based recommendations, thereby reducing the likelihood of errors and ensuring that the advice given is both accurate and appropriate for children. Another approach involves the use of rule-based models, as seen in apps like Woebot, which prioritize safety by avoiding generative AI for user-facing text. Even with rule-based models, there is room for improvement by updating and refining the model parameters. This approach can enhance the user experience while maintaining the safety and reliability of the app. By integrating these strategies, AI techniques in AI-driven mental health apps for children can be better positioned to meet clinical standards and effectively support children’s mental health needs.

### Limitations

It is important to acknowledge the limitations of this study. The first limitation lies in the time-sensitive nature of the app search process. AI-based mental health apps are being developed and launched at an unprecedented rate, reflecting the rapid advancements in AI technology. To mitigate this limitation, we conducted 2 app searches in October 2023 and December 2023. However, this rapid pace of development means that some newly launched apps may not have been captured in our analysis. Future studies could address this limitation by using longitudinal designs with repeated app searches over extended periods to better capture the evolving landscape of AI-based mental health apps. The second limitation concerns the scope of the apps included. Currently, there are very few AI-based mental health apps specifically designed for children available in the market. As a result, we included all AI-based mental health mobile phone apps, including both those explicitly designed for children and those that, while not exclusively targeting children, still consider them part of their intended user base. As AI-based mental health interventions specifically designed for children continue to grow, future research should focus on systematically reviewing apps developed exclusively for children to provide a more targeted analysis. Third, we only included apps available in English, which may have excluded apps offered in other languages. This limitation highlights the need for future studies to explore apps in diverse languages to provide a more inclusive understanding of the global availability and accessibility of AI-based mental health apps for children.

### Conclusion and Implications

AI-powered mobile apps were positioned at the frontier, addressing children’s mental health issues, uniquely combining accessibility and acceptance among the younger demographics. Our study, however, emphasized that the future of these AI-based tools was intrinsically tied to their capacity for significant evolution and enhancement. This included enhanced child-friendly design, clinically impactful outcomes, and superior quality of AI techniques, all of which should be considered nonnegotiable facets of their development. At this juncture, our choices will undeniably carve out the path for AI-powered mental health support for children. It is our collective responsibility as a profession to ensure these digital solutions extend beyond technological sophistication, embodying empathy, effectiveness, and genuine child-centricity. By doing so, we can unlock the full potential of AI, creating a future where every child, regardless of their location or circumstances, has access to robust, reliable, and adequate mental health support.

## Supplementary material

10.2196/58597 Checklist 1 PRISMA (Preferred Reporting Items for Systematic Reviews and Meta-Analyses) checklist.

## Abbreviations

AI: artificial intelligence
MARS: Mobile Application Rating Scale
